# The future of non-viral gene delivery for the treatment of inherited retinal diseases

**DOI:** 10.1016/j.omtn.2022.10.011

**Published:** 2022-11-07

**Authors:** Jyoti Sharma, Eleftherios I. Paschalis

**Affiliations:** 1 Massachusetts Eye and Ear, Department of Ophthalmology, Harvard Medical School, Boston, MA 02114, USA; 2 Schepens Eye Research Institute, Boston Keratoprosthesis Laboratory/Massachusetts Eye and Ear, Harvard Medical School, Boston, MA 02114, USA; 3 Disruptive Technology Laboratory, Massachusetts Eye and Ear, Department of Ophthalmology, Harvard Medical School, Boston, MA 02114, USA

**Keywords:** gene therapy, non-viral, nanoparticles, adeno-associated virus vector, AAV, PEG, retinal diseases

In a recent article in *Molecular Therapy – Nucleic Acids*,^1^ Sun et al. describe a promising therapy for ABCA4-mediated Stargardt disease (STGD). The therapy is based on a non-viral gene delivery platform designed for the treatment of inherited retinal diseases (IRDs) caused by mutations in large genes. This platform allows for the formulation of self-assembling multifunctional lipid nanoparticles loaded with plasmid DNA containing the gene of interest. The authors’ preliminary study using subretinal injections showed that the therapy was safe and effective for short-term gene expression in STGD1 mice.^2^ In the present study, the authors introduce two key modifications in the plasmid sequence to significantly enhance gene expression. This effect was achieved by introducing a gene promoter along with an enhancer in the plasmid sequence. Using repeated subretinal injections in mice, Sun et al. demonstrate dramatic improvement in long-term gene expression without adverse effects. Compared with adenovirus-associated virus (AAV) gene delivery methods, the proposed non-viral method offers improvements in transfection efficiency, immunogenicity, and cargo capacity. However, repeated subretinal injections carry the risk of retinal detachment, an issue that should be addressed in further studies.

IRDs can lead to vision loss or blindness, but gene therapy is poised to ameliorate the deleterious effects of these diseases. Traditionally, viral-based gene delivery methods have been the gold standard of gene therapy. For example, AAV2 was approved by the US Food and Drug Administration for the treatment of type 2 Leber’s congenital amaurosis.^3^ For safety reasons, viral vectors are modified to eliminate immunogenicity and uncontrolled replication after injection. Although viral vectors provide stable, long-term expression of target genes, generating clinical-grade viruses for human use requires complex and laborious processes. Viral vectors also have serious disadvantages, such as low cargo capacity, random integration of genes within the host genome, activation of oncogenes, and inactivation of tumor-suppressor genes. Moreover, viral vectors injected into the retina can enter the brain via uptake by retinal ganglion cells.

The limitations of viral vectors can be potentially overcome by using non-viral delivery methods. For example, synthetic polymers, lipid cations, liposome-based nanoparticles, and polysaccharides show good transfection efficiency. The multifunctional lipid carrier described by Sun et al. is the first to demonstrate release of plasmid DNA or other genetic material through pH-sensitive amphiphilic endosomal escape and reductive cytosolic release. This action is achieved using polyethylene glycol (PEG) to minimize non-specific gene delivery, an approach that enables packaging of large genes with minimal immunogenicity and low risk of random integration into the host genome. In terms of transfection efficiency, the described approach is the same, if not better, than conventional viral gene delivery methods in the treatment of STGD1 mice when multiple subretinal injections are employed.

In the future, non-viral gene delivery systems may be used for the spatiotemporal control of mutant gene/protein expression without the risk of integration into the host genome. Since the retina contains various cell types, cell-specific control by using a promoter or ligand could help improve the specificity and efficacy of non-viral gene therapy. However, some issues must be addressed in pre-clinical studies before the current approach can be applied to humans, namely the risk of immune reactions and complications from the formation of anti-PEG antibodies *in vivo*,^4^ the risk of choroidal neovascularization after PEG injection,^5^ and the risk of retinal detachment following repeated subretinal injections. Despite these limitations, the results presented by Sun et al. are highly encouraging and support the promising future of non-viral gene delivery.
